# The efficacy of transcranial direct current stimulation (tDCS) combined with cognitive behavioural therapy for major depressive disorder: a systematic review and meta-analysis of randomised controlled trials.

**DOI:** 10.1192/j.eurpsy.2026.10614

**Published:** 2026-07-31

**Authors:** O. A. F. S. Da Silva, S. I. D. S. Pereira, A. Mert, D. R. de Aguiar, M. B. Nogueira, J. C. de Araujo, G. A. Prilip, N. N. Sherpa

**Affiliations:** 1 Psycholgy, Universidade Federal do Maranhão - UFMA, São Luís, Brazil; 2 Medicine, University of Buenos Aires, Buenos Aires, Argentina; 3 Psychiatry, Nazilli State Hospital, Aydin, Türkiye; 4 Psycholgy, Universidade Federal de São Paulo - UNIFESP, São Paulo; 5 Medicine, Universidade Federal do Maranhão - UFMA, São Luís; 6 Medicine, Centro Universitário Ingá - Uningá, Maringá; 7 Psychology, Centro Universitário São Camilo, São Paulo, Brazil; 8 Psycholgy, King’s College London, London, United Kingdom

## Abstract

**Introduction:**

Major depressive disorder (MDD) is a prevalent mental health condition, affecting around 10% of the population worldwide. Conventional treatments include psychotherapy, pharmacotherapy, and neuromodulation. Transcranial direct current stimulation (tDCS) is a non-invasive neuromodulation technique that has shown promise in alleviating depressive symptoms. Given evidence that tDCS enhances cognitive control and emotional regulation, it has been hypothesised that combining tDCS with cognitive behavioural therapy (CBT) may yield synergistic therapeutic benefits.

**Objectives:**

To evaluate the efficacy of tDCS combined with CBT in reducing depressive symptoms in patients with MDD, compared with sham tDCS combined with CBT.

**Methods:**

A systematic search of PubMed, Embase, and Cochrane Library databases was conducted in accordance with PRISMA guidelines. The primary outcome was change in depressive symptom severity from pre- to post-intervention. Secondary outcomes included remission and response rates. Data were analysed using Review Manager 5.1.7 (Cochrane Collaboration). Standardised mean differences (SMDs) and risk ratios (RRs) were calculated using random-effects models, with heterogeneity assessed via the I² statistic. Statistical significance was set at p < 0.05.

**Results:**

Five RCTs comprising 173 patients were included; 91 (52.6%) received active tDCS plus CBT. Active tDCS plus CBT did not show a significant advantage over sham tDCS plus CBT for depressive symptom reduction (SMD = –0.14; 95% CI –0.68 to 0.39; p > 0.05; I² = 5%). Likewise, no significant differences were found for remission (RR = 1.49; 95% CI 0.61 to 3.64; p > 0.05; I² = 0%) or response rates (RR = 1.16; 95% CI 0.52 to 2.59; p > 0.05; I² = 4%). The forest plots for the outcomes of reduction in depressive symptoms and response rates can be seen, respectively, in Figures 1 and 2:

**Image 1:**
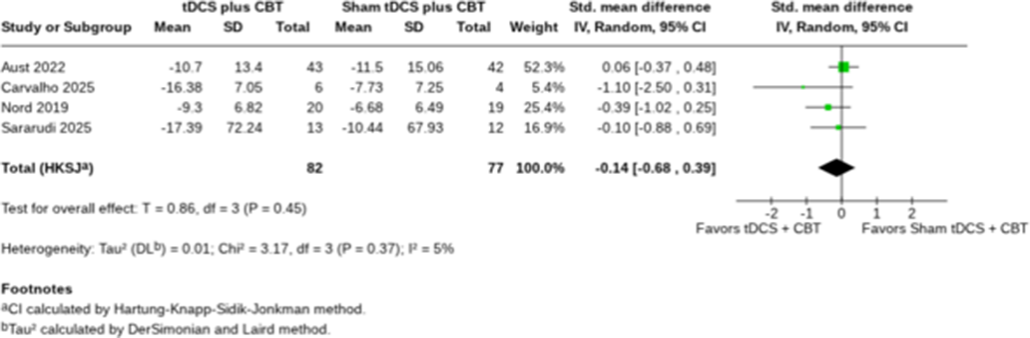
Image 1: Long description.

**Image 2:**
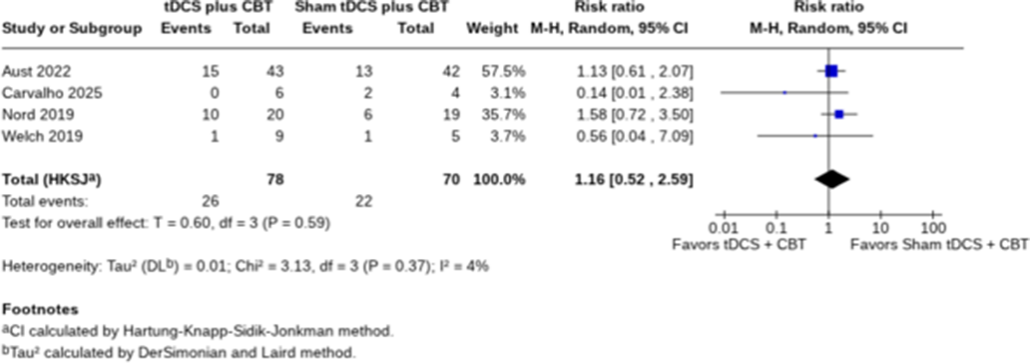
Image 2: Long description.

**Conclusions:**

This meta-analysis suggests that combining tDCS with CBT does not confer additional benefit over sham tDCS with CBT for reducing depressive symptoms or improving remission and response rates in MDD. To our knowledge, this is the first meta-analysis addressing this question. Larger, rigorously designed RCTs are warranted to determine whether tDCS has an adjunctive role alongside psychotherapy.

**Disclosure of Interest:**

None Declared

## Long descriptions

### Image 1: Long description

A forest plot table with columns for Study or Subgroup, t D C S plus C B T (Mean, S D, Total), Sham t D C S plus C B T (Mean, S D, Total), Weight, and Std. mean difference I V, Random, 95% C I.

Individual study data:

* Aust 2022: Weight 52.3%, Std. mean difference 0.06 [-0.37, 0.48].

* Carvalho 2025: Weight 5.4%, Std. mean difference -1.10 [-2.50, 0.31].

* Nord 2019: Weight 25.4%, Std. mean difference -0.39 [-1.02, 0.25].

* Sararudi 2025: Weight 16.9%, Std. mean difference -0.10 [-0.88, 0.69].

The Total (H K S J super a) row shows a combined total of 82 participants in the treatment group and 77 in the sham group, with a pooled Std. mean difference of -0.14 [-0.68, 0.39].

To the right, a graphical forest plot displays green squares for individual studies and a black diamond for the total effect. The x-axis ranges from -2 to 2, where values less than 0 favor t D C S + C B T and values greater than 0 favor Sham t D C S + C B T. The diamond overlaps the zero line, indicating no statistically significant difference.

Statistical text below the plot:

* Test for overall effect: T = 0.86, d f = 3 (P = 0.45).

* Heterogeneity: Tau super 2 (D L super b) = 0.01; Chi super 2 = 3.17, d f = 3 (P = 0.37); I super 2 = 5%.

Footnotes:

a C I calculated by Hartung-Knapp-Sidik-Jonkman method.

b Tau super 2 calculated by DerSimonian and Laird method.

Navigate back to Image 1:.

### Image 2: Long description

A forest plot table with columns for Study or Subgroup, t D C S plus C B T (Events and Total), Sham t D C S plus C B T (Events and Total), Weight, and Risk Ratio M-H, Random, 95% C I.

Individual study data:

* Aust 2022: 15/43 events vs 13/42 events. Weight 57.5%. Risk ratio 1.13 [0.61, 2.07].

* Carvalho 2025: 0/6 events vs 2/4 events. Weight 3.1%. Risk ratio 0.14 [0.01, 2.38].

* Nord 2019: 10/20 events vs 6/19 events. Weight 35.7%. Risk ratio 1.58 [0.72, 3.50].

* Welch 2019: 1/9 events vs 1/5 events. Weight 3.7%. Risk ratio 0.56 [0.04, 7.09].

Summary statistics:

* Total (H K S J super a): 78 total in treatment group, 70 in sham group. Total events: 26 vs 22. Weight 100.0%. Pooled Risk Ratio 1.16 [0.52, 2.59].

* Test for overall effect: T = 0.60, d f = 3 (P = 0.59).

* Heterogeneity: Tau super 2 (D L super b) = 0.01; Chi super 2 = 3.13, d f = 3 (P = 0.37); I super 2 = 4%.

The graphical forest plot on the right features a horizontal axis ranging from 0.01 to 100 on a log scale, with 1 as the line of no effect. Values to the left favor t D C S + C B T and values to the right favor Sham t D C S + C B T. Individual studies are represented by blue squares with horizontal error bars. The pooled result is represented by a black diamond centered at 1.16, crossing the line of no effect.

Navigate back to Image 2:.

